# CH_4_ Synthesis from CO_2_ and H_2_O of an Electron Source over Rh–Ru Cocatalysts Loaded on NaTaO_3_:Sr Photocatalysts

**DOI:** 10.1021/jacs.3c06413

**Published:** 2023-08-21

**Authors:** Wasusate Soontornchaiyakul, Shunya Yoshino, Tomoki Kanazawa, Rie Haruki, Dongxiao Fan, Shunsuke Nozawa, Yuichi Yamaguchi, Akihiko Kudo

**Affiliations:** † Department of Applied Chemistry, Faculty of Science, Tokyo University of Science, 1-3 Kagurazaka, Shinjuku-ku, Tokyo 162-8601, Japan; ‡ Institute of Materials Structure Science, High Energy Accelerator Research Organization, Tsukuba, Ibaraki 305-0801, Japan; § Carbon Value Research Center, Research Institute for Science & Technology, Tokyo University of Science, 2641 Yamazaki, Noda-shi, Chiba-ken 278-8510, Japan

## Abstract

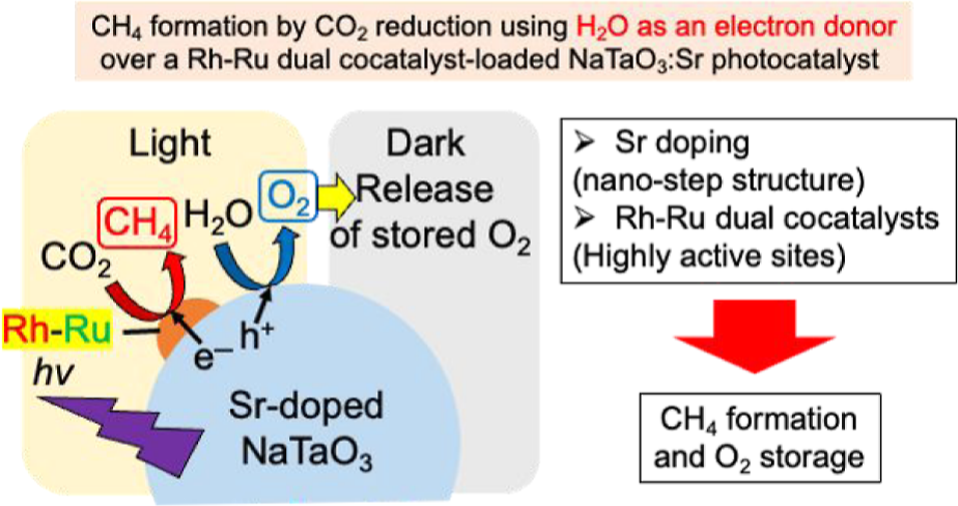

CO_2_ reduction as an artificial photosynthetic system is a promising technology to produce green energies and chemicals because it uses light energy to convert H_2_O and CO_2_ into valuable products such as CO, HCOOH, CH_3_OH, CH_4_, and preferably higher hydrocarbons. In photocatalytic reduction, water should be used as hydrogen and electron sources for CO_2_ reduction. Moreover, CH_4_ formation is an attractive and challenging topic because of the eight-electron-reducing product of CO_2_. Herein, we report the development of a new Rh–Ru cocatalyst decorated on an alkaline earth-doped NaTaO_3_ surface for the photocatalytic CO_2_ reduction to form CH_4_ using water as an electron donor. CH_4_ was obtained by a photocatalytic “uphill” reaction of CO_2_ reduction using Rh–Ru cocatalyst-loaded NaTaO_3_:Sr, water, and CO_2_ in an aqueous suspension system. About 10% of a selectivity (electronic efficiency) was obtained for CH_4_ formation under ambient conditions accompanied with O_2_ evolution of the oxidation product of H_2_O.

## Introduction

Photocatalytic CO_2_ reduction as artificial photosynthesis has attracted attention as a chemical technology for CO_2_ fixation aiming at a sustainable society. In artificial photosynthesis, solar energy and CO_2_ are directly converted to useful chemical products. Moreover, the reaction can be operated under ambient temperature and pressure, and abundant water functions as hydrogen and electron sources.^1,2^ To achieve photocatalytic CO_2_ reduction as artificial photosynthesis being an uphill reaction (Δ*G* > 0), CO_2_ reduction products must be obtained accompanied with reasonable O_2_ evolution by water oxidation as the counterpart but not using strong sacrificial electron donors. Moreover, employing semiconductor photocatalyst powders is desirable because of their simplicity for practical use.^3^ Recently, efficient and selective CO formation as two-electron-photocatalytic CO_2_ reduction has been reported since discovering a Ag cocatalyst even using a photocatalyst powder-dispersion system.^1,4−8^ On the other hand, photocatalytic CH_4_ formation is still a challenging topic because of a tough reaction accompanied with eight-electron reduction. Selective hydrocarbon formation such as CH_4_ has been achieved by electrochemical CO_2_ reduction using a Cu electrode under ambient condition^9−12^ and Fe, Co, and Ni electrodes under high pressure of CO_2_.^13,14^ In contrast, although there are a lot of reports to produce CH_4_ over semiconductor photocatalysts, mainly TiO_2_, by loading various cocatalysts such as Cu, Ru, and Rh, the amount of evolved CH_4_ is quite small compared with the amount of an employed photocatalyst, and no O_2_ evolution is observed.^15−18^ There are only a few papers in which an amount of O_2_ evolved was determined, though the activity was low.^19−21^ It is challenging to obtain a reasonable amount of CH_4_ of an eight-electron reduction product with a stoichiometric O_2_ evolution by photocatalytic CO_2_ reduction using water as an electron donor without any strong electron donors. The critical issue comes from “lack of an active site to convert CO_2_ to CH_4_ relating to eight photogenerated-electrons and hydrogens” and “low photocatalytic activity”. Moreover, the importance of the present research is to use water as an electron donor to reduce CO_2_ to CH_4_ accompanied with O_2_ evolution. For breaking through the present stage, it is indispensable to develop a new cocatalyst to convert CO_2_ to CH_4_ accompanied by obvious O_2_ evolution on a highly efficient photocatalyst. We have developed a NaTaO_3_:Sr photocatalyst showing high activity for water splitting and CO_2_ reduction to form CO using water as an electron donor.^6,22^ From such background, in the present report, we developed a new Rh–Ru cocatalyst and demonstrated photocatalytic CH_4_ formation using water as an electron donor and artificial photosynthesis using the NaTaO_3_:Sr photocatalyst.

## Experimental Section

### Photocatalyst Preparation

Doped and non-doped NaTaO_3_ powders were prepared by a solid-state reaction. The starting materials of Na_2_CO_3_ (Kanto Chemical; 99.8%), Ta_2_O_5_ (rare metallic; 99.99%), CaCO_3_ (Kanto Chemical; 99.5%), SrCO_3_ (Kanto Chemical; 99.9%), BaCO_3_ (Kanto Chemical; 99.0%), and La_2_O_3_ (Kanto Chemical; 99.99%) were mixed and grinded in an alumina mortar. A molar ratio of Na/Sr/Ta as 1.0395:0.01:1 for 1% of Sr-doping. An excess amount of Na (5 mol %) was required to compensate a volatilization. The mixtures were put in a Pt crucible and calcined at 1173 K for an hour. After being cooled to room temperature, the mixtures were grinded again in the mortar and calcined again at 1423 K for 10 h using a Pt crucible.

Rh and Ru cocatalysts were loaded on photocatalysts by a liquid-phase chemical reduction method using RhCl_3_ (Tanaka Kikinzoku, 36–39.08% as Rh in RhCl_3_·3H_2_O) and RuCl_4_ (Tanaka Kikinzoku, 36% as Ru in RuCl_4_·*n*H_2_O) as cocatalyst sources and NaPH_2_O_2_ (Kanto Chemical; 82.0–86.5% as NaPH_2_O_2_ in NaPH_2_O_2_·H_2_O) as a reducing reagent. A mixed aqueous RhCl_3_ and RuCl_4_ solution was added into an aqueous suspension containing the photocatalyst powder and stirred at 323 K for 2 h. The aqueous NaPH_2_O_2_ solution was added to the mixture matured by stirring at 343 K for 12 h. The amount of NaPH_2_O_2_ was set as 40 times more molar quantity against Rh and Ru sources. The obtained Rh–Ru cocatalyst-loaded photocatalysts were washed with water and dried overnight at room temperature.

X-ray diffraction (Rigaku; MiniFlex600) was used to confirm a single phase of obtained NaTaO_3_:M (M = non, Ca, Sr, Ba, and La). Surface components of Rh–Ru/NaTaO_3_:Sr were analyzed by X-ray photoelectron spectroscopy (XPS; JEOL; JPS-9010MC) with a Mg K_α_ anode. Rh–Ru/NaTaO_3_:Sr powder before and after photocatalytic CO_2_ reduction was loaded on a carbon tape for the XPS measurement. The loaded amounts of cocatalysts were also measured by X-ray fluorescence (XRF; Rigaku; NEXDE). Morphologies of photocatalysts and loaded cocatalysts were observed by transmission electron microscopy (TEM; JEOL; JEM-2100F). X-ray absorption near-edge structure (XANES) of Ru and Ru K-edge were measured at AR-NW10A of the Photon Factory Advanced Ring in Tsukuba, Japan. The incident X-ray was monochromatized by a Si(311) double-crystal monochromator.

### Photocatalytic Reaction

Photocatalytic CO_2_ reduction reaction and water splitting were conducted using a gas-flow system equipped with an inner irradiation quartz cell. Photocatalyst powder (1.5 g) was dispersed in 350 mL of water without any additives. CO_2_ or Ar gas (1 atm) was bubbled into the aqueous suspension at a flow rate of 30 mL min^–1^ for photocatalytic CO_2_ reduction and water splitting, respectively. A 400 W high-pressure Hg lamp (SEN, HL-400EH-5) was employed as a light source. Amounts of evolved H_2_, O_2_, CO, and CH_4_ were determined using online gas chromatographs (Shimadzu GC-8A equipped with the MS-5A column, a TCD, and an Ar carrier for H_2_ and O_2_; Shimadzu GC-8A equipped with the MS-13X column, a FID with methanizer, and an Ar carrier for CO and CH_4_). The selectivity for CH_4_ formation, the stored O_2_ ratio, the reacted electron to hole ratio (e^–^/h^+^), and the turnover number (TON) were calculated according to following equations.
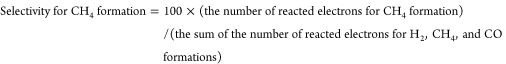
1
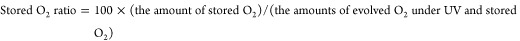
2
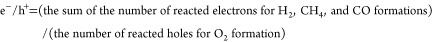
3
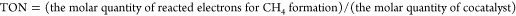
4

## Results and Discussion

**Figure 1:**
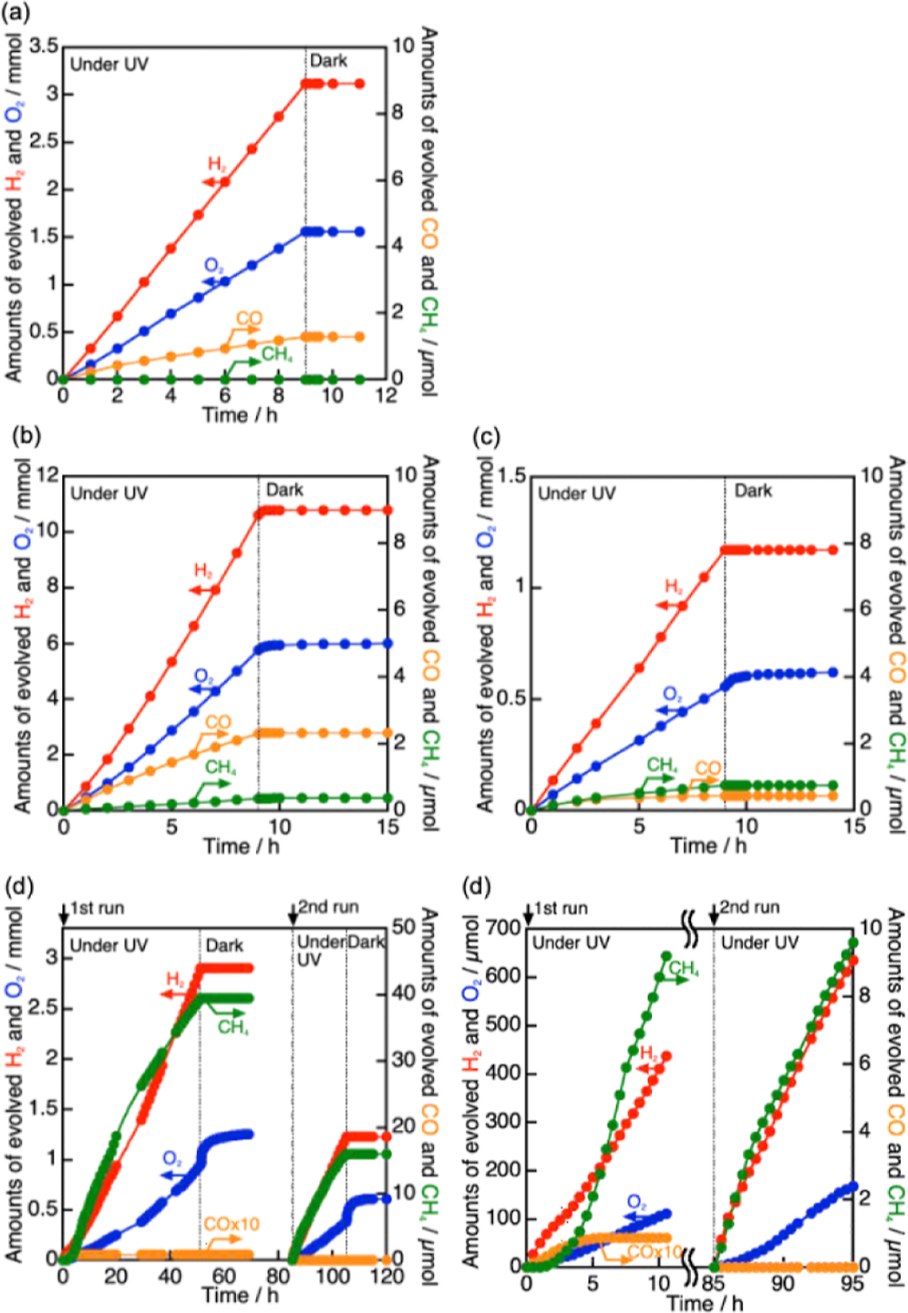
Photocatalytic CO_2_ reduction using (a) NaTaO_3_:Sr, (b) Ru(0.375 wt %)/NaTaO_3_:Sr(1%), (c) Rh(0.5 wt %)/NaTaO_3_:Sr(1%), and (d) Rh(0.5 wt %)–Ru(0.375 wt %)/NaTaO_3_:Sr(1%) photocatalysts under UV irradiation. Photocatalyst: 1.5 g, solution: water without any additives (350 mL, pH 4–5), flow gas: CO_2_ (1 atm), light source: 400 W high pressure Hg lamp, and cell: inner-irradiation quartz cell.

We conducted photocatalytic CO_2_ reduction using Rh(0.5 wt %)–Ru(0.375 wt %) cocatalyst-loaded NaTaO_3_:Sr(1%) in an aqueous solution bubbling 1 atm of CO_2_, as shown in Figure 1 and Table 1. In the present experiment, the released gases from the suspension in the flow reactor were quantified even under dark after light irradiation was stopped because the released O_2_ after the photocatalytic reaction was observed, as shown in Figure 1. The amount of released O_2_ under dark after stopping UV irradiation was set at “stored O_2_” columns in Table1.

**Table 1:** Effects of Rh–Ru Cocatalysts and Alkaline Earth Metal Dopants on Photocatalytic CO_2_ Reduction Using NaTaO_3_:Sr(1%)^a^

entry	photocatalyst	cocatalyst	rates of gas evolution under UV/μmol h^–1^				CH_4_ select %	amounts of evolved gases/μmol (9 h)					stored O_2_ ratio %	e^–^/h^+^
			H_2_	CO	CH_4_	O_2_		H_2_	CO	CH_4_	O_2_	stored O_2_		
1	NaTaO_3_:Sr(1%)	none	350	0.12	0	177	0	3118	1.29	0	1564	0	0	1.0
2	NaTaO_3_:Sr(1%)	Ru	1339	0.20	0.04	737	0.01	10 603	2.31	0.36	5761	242	4.0	0.9
3	NaTaO_3_:Sr(1%)	Rh	129	0.02	0.07	60	0.22	1171	0.44	0.76	557	62	10	1.0
4	NaTaO_3_:Sr(1%)	Rh–Ru	54	0	1.44	14	9.64	443	0.08	9.10	130	44	25	1.4
5	NaTaO_3_	none	139	0.10	0	65	0	1186	1.02	0	527	0	0	1.1
6	NaTaO_3_	Rh–Ru	20	0.03	0.11	8	2.12	222	0.23	0.82	79	7	8.1	1.3

a Photocatalyst: 1.5 g, cocatalyst: Ru (0.375 wt %) and Rh (0.5 wt %) loaded by a liquid phase reduction, solution: water without any additives (350 mL, pH 4–5), flow gas: CO_2_ (1 atm, 30 mL min^–1^), light source: 400 W high pressure Hg lamp, cell: inner-irradiation quartz cell, and total irradiation time: 9 h. Rates of gas evolution were estimated after induction period of UV irradiation. CH_4_ selectivity was calculated using the rates of gas evolution. e^–^/h^+^ was calculated from amounts of evolved gases including stored O_2_.

Bare NaTaO_3_:Sr split water into stoichiometric amounts of H_2_ and O_2_, but gave quite low activity for CO_2_ reduction (Figure 1a; Table 1 entry 1), because non-loaded NaTaO_3_:Sr did not have an effective active site for CO_2_ reduction.^6^ On the other hand, a Ru cocatalyst enhanced not only water splitting but also CO_2_ reduction to form a small amount of CH_4_ (Figure 1b; Table 1 entry 2). A Rh cocatalyst slightly enhanced CH_4_ formation compared with the Ru cocatalyst, though water splitting was suppressed (Figure 1c; Table 1 entry 3), meaning that the Rh cocatalyst functioned as an active site for CH_4_ formation by reducing CO_2_. Coloading of Rh and Ru much enhanced the CH_4_ formation (Figure 1d; Table 1 entry 4). The enhancement of CH_4_ formation might be due to the efficient supply of adsorbed hydrogen and photogenerated electron from Ru to Rh which should be a critical issue for reducing CO_2_ to form CH_4_ being multi-proton and electron reaction. We also confirmed that formic acid was not detected with an ion chromatograph. Alcohol and formaldehyde compounds should not be stored in the reactant solution because they are smoothly oxidized by photogenerated holes in a photocatalyst as a sacrificial reagent. We also investigated the effect of loading amounts of Rh–Ru cocatalysts on photocatalytic CO_2_ reduction using NaTaO_3_:Sr(1%), as shown in Table 2. When the ratio of Rh to Ru was fixed and the loading amounts were varied, Rh(0.5 wt %)–Ru(0.375 wt %) was optimal for CH_4_ formation (entries 5–10). Then, when the amount of Ru was changed from 0 to 0.75 wt % with 0.5 wt % of Rh (entries 3, 4, 7, 11, and 12), the highest activity for CH_4_ formation was obtained by loading 0.375 wt % of Ru. Thus, the effect of the cocatalyst on CH_4_ formation was in order of Ru < Rh ≪ Rh–Ru. The apparent quantum yield was estimated to be about 0.016% at 270 nm for CH_4_ formation on the optimized photocatalyst judging from the efficiency for water splitting on a NiO/NaTaO_3_:La photocatalyst.^23^

**Table 2:** Effect of Loading Amounts of Rh–Ru Cocatalysts on Photocatalytic CO_2_ Reduction Using NaTaO_3_:Sr(1%)^a^

entry	loaded Rh/μmol (wt %)	loaded Ru/μmol (wt %)	Rh/Ru	rates of gas evolution under UV/μmol h^–1^				CH_4_ select %	amounts of evolved gases/μmol (9 h)					stored O_2_ ratio %	e^–^/h^+^
				H_2_	CO	CH_4_	O_2_		H_2_	CO	CH_4_	O_2_	stored O_2_		
1	0	0		350	0.12	0	177	0	3118	1.29	0	1564	0	0	1.0
2	0	54.9 (0.375)		1339	0.20	0.04	737	0.01	10 603	2.31	0.36	5761	242	4.0	0.9
3	72.9 (0.5)	0		129	0.02	0.07	60	0.22	1171	0.44	0.76	557	62	10.0	0.9
4	72.9 (0.5)	37.2 (0.25)	1.96	60	0	0.72	17	4.58	434	trace	4.02	119	89	42.9	1.1
5	29.8 (0.2)	22.5 (0.15)	1.32	75	0.01	0.50	25	2.60	568	0.09	4.11	177	100	36.1	1.1
6	44.2 (0.3)	32.3 (0.225)	1.37	74	0.01	0.65	24	3.39	519	0.09	4.73	163	66	28.8	1.2
7	72.0 (0.5)	55.1 (0.375)	1.31	54	0	1.44	14	9.64	443	0.08	9.10	130	44	25.3	1.4
8	101.8 (0.6)	76.5 (0.45)	1.33	44	0	1.26	10	10.28	340	trace	7.06	80	61	43.3	1.3
9	144.8 (1)	110.6 (0.75)	1.31	41	0	1.01	9.8	8.97	404	trace	4.43	100	60	37.5	1.3
10	363.9 (2.5)	271.1 (1.87)	1.34	75	0.02	0.23	19	1.21	1046	0.31	1.77	245	89	26.6	1.6
11	72.6 (0.5)	74.0 (0.5)	0.98	54	trace	1.00	14	6.90	423	0.09	4.84	118	64	35.2	1.2
12	72.8 (0.5)	110.0 (0.75)	0.66	61	trace	0.66	15	4.15	393	0.06	2.76	93	72	43.6	1.2
13	30.1 (0.2)	56.2 (0.375)	0.54	835	0.03	0.26	394	0.12	4694	0.19	1.40	2105	139	6.2	1.0

a Photocatalyst: 1.5 g, cocatalyst: a liquid phase reduction (loaded amounts (/μmol) was determined by XRF), solution: water without any additives (350 mL, pH 4–5), flow gas: CO_2_ (1 atm, 30 mL min^–1^), light source: 400 W high pressure Hg lamp, cell: inner-irradiation quartz cell, and total irradiation time: 9 h. Rates of gas evolution were estimated after induction period of UV irradiation. CH_4_ selectivity was calculated using the rates of gas evolution. e^–^/h^+^ was calculated from amounts of evolved gases including stored O_2_.

In the photocatalytic CO_2_ reduction using the Rh–Ru/NaTaO_3_:Sr photocatalyst (Figure 1d′), small amounts of CO and CH_4_ were obtained accompanied with H_2_ and O_2_ evolved by water splitting in the induction period for 5 h of UV light irradiation. CH_4_ evolution gradually increased through the induction period, while CO evolution was suppressed. After that, the Rh–Ru/NaTaO_3_:Sr(1%) steadily produced CH_4_, H_2_, and O_2_. Gradual activation for CH_4_ formation was not observed in the second run being different from the induction period of the first run, indicating that the cocatalyst changed during the induction period of the first run. After light irradiation was stopped at 51 h, obvious O_2_ was released from the suspension for several hours under dark, while H_2_, CO, and CH_4_ were not so (Figure S1). In other words, a part of O_2_ was stored in the suspension under UV light irradiation, and the stored O_2_ was quickly released by stopping light irradiation. The O_2_ release was observed even 17 h after stopping UV light irradiation. The O_2_ storage behavior was also observed for Ru- and Rh-loaded NaTaO_3_:Sr (Table 1 entries 2 and 3), whereas it was not so over non-loaded NaTaO_3_:Sr (Table 1 entry 1). Therefore, loading of the Rh–Ru cocatalyst should be essential for the O_2_ storage behavior. The O_2_ storage was also seen for the second run, as shown in Figure 1d.

The e^–^/h^+^ estimated from the amounts of evolved gasses including stored O_2_ in the second run of Figure 1d was 1.05 being almost unity. The TON of the molar quantity of the reacted electrons for CH_4_ formation in the 1st + 2nd runs to the loaded cocatalyst was estimated to be 3.5. We also conducted a control experiment under Ar flow using the Rh–Ru/NaTaO_3_:Sr photocatalyst at pH 4 adjusted by H_2_SO_4_ because pH of water saturated with CO_2_ under 1 atm was 4 (Figure S2). CO and CH_4_ did not evolve under the Ar atmosphere, revealing that the origin of CO and CH_4_ obtained in Figure 1d was flowed CO_2_. In contrast to the CH_4_ formation, the O_2_ storage was observed under not only CO_2_ but also an Ar atmosphere in which water splitting proceeded (Figure S2). Thus, the Rh–Ru/NaTaO_3_:Sr(1%) photocatalyst has unique property to produce CH_4_ using water as an electron donor and store O_2_ under UV light irradiation.

We examined the effect of Sr dopants into the NaTaO_3_ photocatalyst on CH_4_ formation and O_2_ storage ability. Bare NaTaO_3_ did not show CH_4_ formation and O_2_ storage abilities. When the Rh–Ru cocatalyst was loaded, NaTaO_3_ without Sr dopant showed low activities for CH_4_ formation and O_2_ storage (Table 1 entries 5 and 6). In contrast to them, Sr doping much enhanced CH_4_ formation and O_2_ storage with the loading of Rh–Ru cocatalysts (entries 4 and 6). We have also investigated the effect of other dopants on CO_2_ reduction over NaTaO_3_:M (M = Ca, Sr, Ba, and La) loaded with/without the Rh–Ru cocatalysts, as shown in Table 3. All dopants gave CH_4_ formation and O_2_ storage when the Rh–Ru cocatalyst was loaded. Sr was the most effective dopant. We have reported that those dopants formed nano-step structures on the surface of a NaTaO_3_ photocatalyst particle and widened the band gap from 4.0 to 4.1 eV, resulting in enhancement of water splitting.^22,23^ Therefore, it is concluded that a synergetic effect by loading the Rh–Ru cocatalyst and Sr doping to create surface nano-step structure was a key factor for reasonable CH_4_ formation and O_2_ storage.

**Table 3:** Effects of Dopant M on Photocatalytic CO_2_ Reduction Using Rh–Ru/NaTaO_3_:M^a^

entry	photocatalyst	surface step structure	cocatalyst	rates of gas evolution under UV/μmol h^–1^				CH_4_ select %	amounts of evolved gases/μmol (9 h)					stored O_2_ ratio %	e^–^/h^+^
				H_2_	CO	CH_4_	O_2_		H_2_	CO	CH_4_	O_2_	stored O_2_		
1	NaTaO_3_	absent	none	139	0.10	0	65	0	1186	1.02	0	527	0	0	1.1
2	NaTaO_3_	absent	Rh–Ru	20	0.03	0.11	8	2.15	222	0.23	0.82	79	7	8.1	1.3
3	NaTaO_3_:Ca(1%)	incomplete	none	233	0.09	0.01	115	0.02	1951	0.08	0.97	960	0	0	1.0
4	NaTaO_3_:Ca(1%)	incomplete	Rh–Ru	44	0.01	0.8	12	6.78	312	0.1	3.9	84	82	49.4	1.0
5	NaTaO_3_:Sr(0.5%)	absent	Rh–Ru	39	0.01	0.81	11	7.67	319	0.1	3.8	85	48	36.1	1.3
6	NaTaO_3_:Sr(1%)	incomplete	none	350	0.12	0	177	0	3118	1.29	0	1564	0	0	1.0
7	NaTaO_3_:Sr(1%)	incomplete	Rh–Ru	54	0	1.44	14	9.64	443	0.08	9.10	130	44	25.3	1.4
8	NaTaO_3_:Sr(2%)	complete	Rh–Ru	41	0.01	1.3	12	11.25	351	0.05	6.77	77	79	50.6	1.2
9	NaTaO_3_:Sr(5%)	complete	Rh–Ru	33	0.01	1.1	9	11.76	299	0.05	6.49	70	86	55.1	1.0
10	NaTaO_3_:Ba(1%)	incomplete	none	233	0.04	0	114	0	2105	0.95	0	1015	0	0	1.0
11	NaTaO_3_:Ba(1%)	incomplete	Rh–Ru	12	0.01	0.23	4	7.12	150	0.15	1.4	36	26	41.9	1.3
12	NaTaO_3_:La(1%)	complete	none	483	0.09	0	228	0	4362	1.04	0	2094	0	0	1.04
13	NaTaO_3_:La(1%)	complete	Rh–Ru	52	0.01	0.5	15	3.7	419	0.12	4.33	131	73	35.7	1.05

a Photocatalyst: 1.5 g, cocatalyst: Ru (0.375 wt %) and Rh (0.5 wt %) loaded by a liquid phase reduction, solution: water without any additives (350 mL, pH 4–5), flow gas: CO_2_ (1 atm, 30 mL min^–1^), light source: 400 W high pressure Hg lamp, cell: inner-irradiation quartz cell, and total irradiation time: 9 h. Rates of gas evolution were estimated after an induction period of UV irradiation. CH_4_ selectivity was calculated using the rates of gas evolution. e^–^/h^+^ was calculated from amounts of evolved gases including stored O_2_.

The O_2_ storage might be due to adsorption of O_2_ molecule or intermediate of evolved O_2_ on the surface of Rh–Ru/NaTaO_3_ with and without dopant. The e^–^/h^+^ ratio estimated from the gas evolution rates approached 1 with an increase in the photocatalytic reaction time, as shown in Figures 1d and S1, implying that the stored O_2_ was gradually saturated. The molar quantity of stored O_2_ molecule (310 μmol) was larger than those of Ru (55.6 μmol) and Rh (72.9 μmol) calculated from first run of Figure 1d, indicating that the O_2_ storage occurred on not only the cocatalyst but also photocatalyst surface. Although photoadsorption of O_2_ would be one possibility, the adsorbed O_2_ should immediately have desorbed. Such slow release of O_2_ was not observed, when O_2_ was flowed in the suspension of the photocatalyst. Moreover, O_2_ adsorption on the Rh–Ru cocatalyst should not be a major process because the cocatalyst is a reduction site to form H_2_ and CH_4_, and the adsorbed O_2_ should easily have been reduced. We conducted the photocatalytic reaction using Rh–Ru/NaTaO_3_:Sr by flowing the mixture of CO_2_ and O_2_. However, the CH_4_ production rate was not improved by the addition of O_2_. However, the positive relationship between appearance of CH_4_ formation and the O_2_ storage is actually observed. No O_2_ storage induced no CH_4_ formation. Although formation of other oxidation product such as H_2_O_2_ and percarbonate in the reactant solution would be another possibility, they were not clearly detected. Therefore, we cannot conclude the firm mechanism for O_2_ storage at present. However, the cocatalyst is necessary to realize O_2_ storage, and then, synergetic effect or interaction between the cocatalyst and the photocatalyst should affect the O_2_ storage ability.

**Figure 2:**
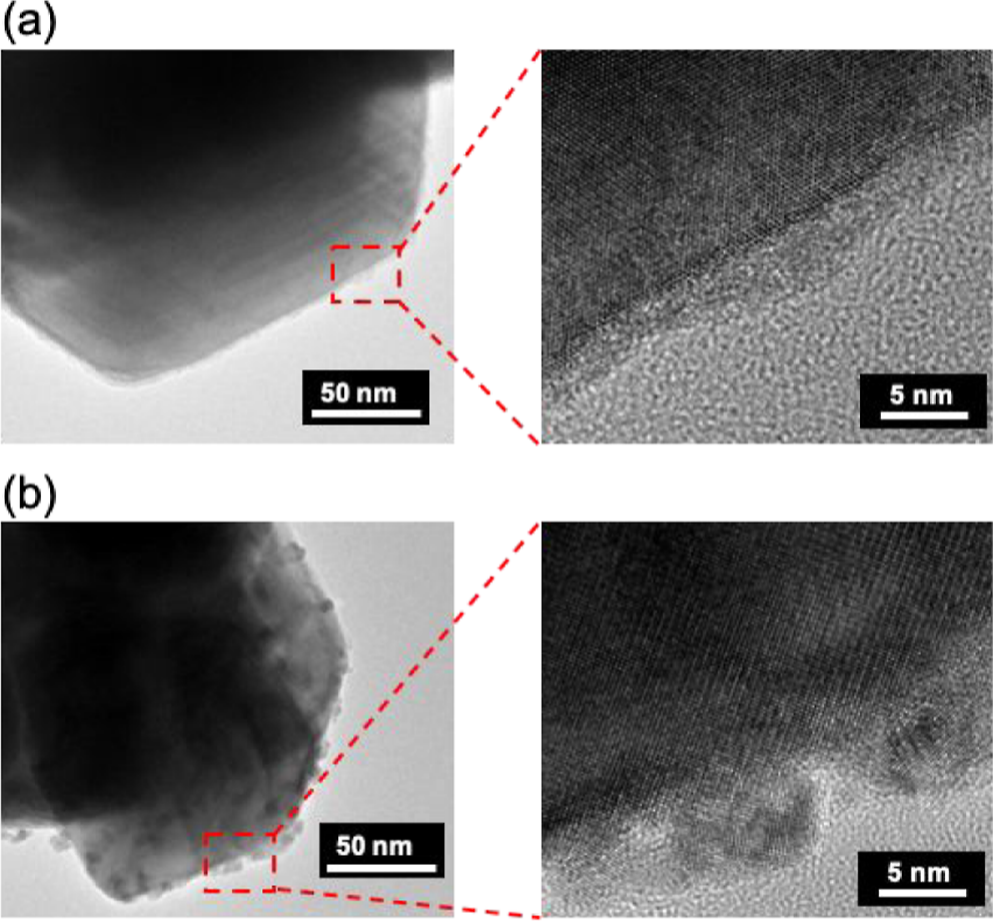
TEM images of Rh(0.5 wt %)–Ru(0.375 wt %) cocatalyst-loaded NaTaO_3_:Sr (1%) photocatalyst (a) before and (b) after photocatalytic CO_2_ reduction (9 h).

Rh–Ru/NaTaO_3_:Sr(1%) powders before and after CO_2_ reduction were characterized to reveal how the cocatalyst functioned. Figure 2 shows TEM images of the Rh–Ru cocatalyst loaded on NaTaO_3_:Sr(1%) before and after CO_2_ reduction by TEM (JEOL; JEM-2100F). A clear particle was not observed on NaTaO_3_:Sr before CO_2_ reduction in the present resolution, while there were aggregated particles after CO_2_ reduction. Although we would have checked the cocatalyst using TEM–EDS before and after photocatalytic CO_2_ reduction, unfortunately, we could not distinguish Ru from Rh nanoparticles because of limitation of energy and spatial resolution. It was not clear whether the Rh and Ru made an alloy or were separately deposited. The surface composition of Rh–Ru/NaTaO_3_:Sr(1%) was analyzed by XPS (JEOL; JPS-9010MC) with a Mg K_α_ anode, as shown in Table 4. The area ratios of Rh and Ru to Ta of the sample after CO_2_ reduction were smaller than those before CO_2_ reduction though the total amounts of Rh and Ru in these samples did not change before and after the reaction judging from XRF (Rigaku; NEXDE). The decrease in the area ratios of Rh/Ta and Ru/Ta that indicated relative coverage by the cocatalysts to the surface of the NaTaO_3_:Sr photocatalyst supported aggregation of the loaded cocatalysts during photocatalytic CO_2_ reduction. Dissolution of the cocatalyst by photooxidation and subsequent deposition by photoreduction and/or migration of the metal atoms on the surface of photocatalyst would induce the aggregation. Similar aggregation by photooxidation and subsequent deposition by photoreduction was observed for a Ag cocatalyst on the BaLa_4_Ti_4_O_15_ photocatalyst for CO_2_ reduction.^4^ Moreover, a larger area ratio of Ru/Rh in XPS through CO_2_ reduction indicated Ru-rich surface (Rh is buried) or more aggregation of Rh than Ru. Although the area ratio significantly changed even after 1 h of light irradiation, the change still occurred slightly judging from the result at 9 h. This alternation would bring the induction period of the CO_2_ reduction to form CH_4_ and CO, as shown in Figure 1d′.

**Table 4:** Elemental Analysis of XPS and XRF for a Rh(0.5 wt %)–Ru(0.375 wt %) Cocatalyst-Loaded NaTaO_3_:Sr(1%) Photocatalyst^a^

CO_2_ reduction	peak area ratio from XPS			molar ratio from XRF		
	Rh/Ta	Ru/Ta	Ru/Rh	Rh/Ta	Ru/Ta	Ru/Rh
before	1.74	0.52	0.30	0.012	0.0093	0.76
after 1 h	0.30	0.28	0.94			
after 9 h	0.14	0.18	1.24	0.012	0.0092	0.77

a Area of Rh 3d (Rh 3d_3/2_ + Rh 3d_5/2_), Ru 3p (Ru 3p_1/2_ + Ru 3p_3/2_), and Ta 4d (Ta 4d_3/2_ + Ta 4d_5/2_) were used for calculation of an area ratio. Rh(0.5 wt %)–Ru(0.375 wt %) corresponds to Rh(1.22 mol %)–Ru(0.94 mol %) to Ta.

**Figure 3:**
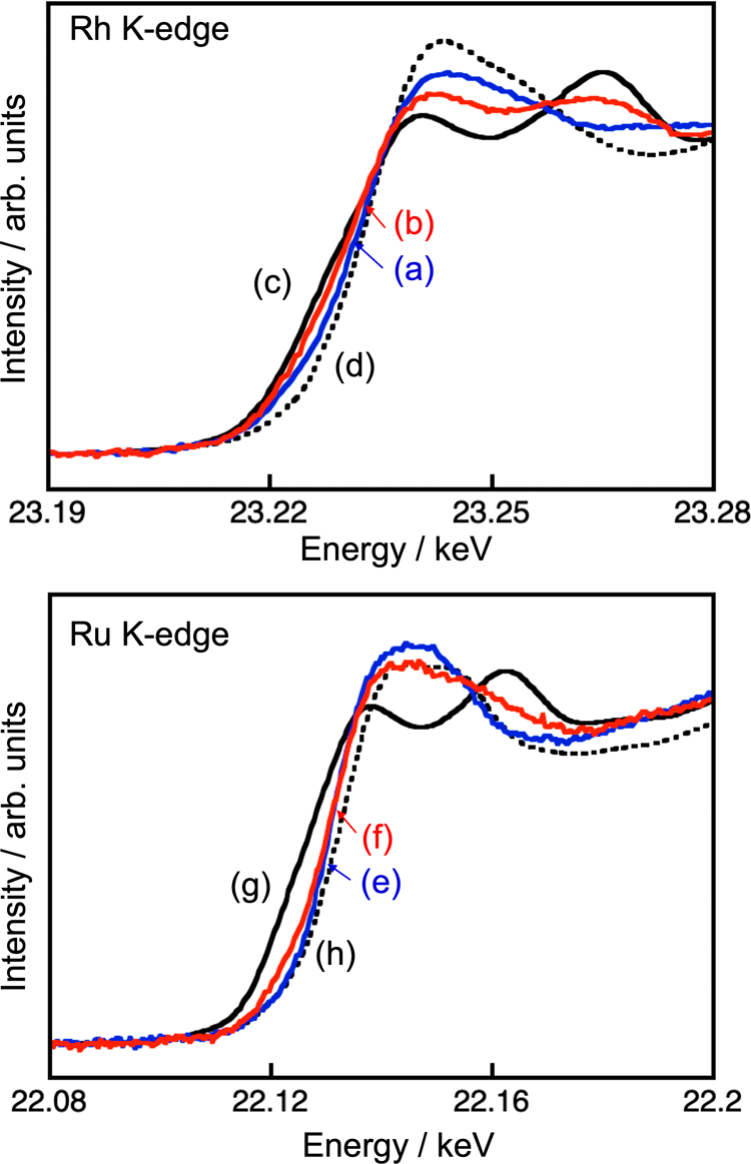
Rh and Ru K-edge XANES spectra of Rh(0.5 wt %)–Ru(0.375 wt %) cocatalyst-loaded NaTaO_3_:Sr(1%) photocatalyst (a,e) before and (b,f) after photocatalytic CO_2_ reduction (9 h). (c) Rh metal, (d) Rh_2_O_3_, (g) Ru metal, and (h) RuO_2_ were measured as a reference sample.

The chemical states of the loaded Rh and Ru were analyzed by XANES for each K-edge using AR-NW10A of the photon factory advanced ring, as shown in Figure 3. The Rh before CO_2_ reduction was loaded as a mixture of oxidized Rh (and/or hydrolyzed Rh) and metallic Rh judging from the position of the absorption edge and the shape of the post edge (Figure 3a), even if NaPH_2_O_2_ was used as a reducing reagent in the present loading step. By carrying out the photocatalytic CO_2_ reduction, the absorption edge shifted to low energy direction, and the shape of the post edge changed to metallic Rh, but a part of oxidized Rh remained. The change of the chemical state would occur due to dissolution of the cocatalyst by photooxidation and subsequent position by photoreduction during the photocatalytic CO_2_ reduction, as mentioned in the previous paragraph. The Ru on NaTaO_3_:Sr before CO_2_ reduction was also a mixture of the oxide and metal. However, in the case of Ru, the shift to low energy direction of the absorption edge and the change of the shape of the post edge was small even after CO_2_ reduction, suggesting that Ru was mainly maintained as oxidized (and/or hydrolyzed) state being different from Rh. These particle size and chemical state alternation of loaded cocatalysts should contribute to the induction period observed in the first run of photocatalytic CO_2_ reduction (Figure 1d′). Then, metallic Rh + hydrolyzed and/or oxidized Rh and Ru particles with aggregation functioned as a cocatalyst for CO_2_ reduction on a NaTaO_3_:Sr photocatalyst.

## Conclusions

We successfully demonstrated photocatalytic CO_2_ reduction to form CH_4_ using water as an electron donor by using Rh–Ru/NaTaO_3_:Sr. It is stressed that reasonable O_2_ evolution by water oxidation was observed as the counterpart of CO_2_ reduction to CH_4_, indicating that the uphill reaction proceeded photocatalytically. The present Rh–Ru cocatalyst led not only conversion of CO_2_ to CH_4_ but also unique O_2_ storage behavior. The Rh–Ru cocatalyst and Sr doping are necessary for the reasonable CH_4_ formation and O_2_ storage. The combination of strategies for loading cocatalysts and doping was a key factor to realize the reasonable CH_4_ formation.

## Supporting Information Available

Production rate for photocatalytic CO_2_ reduction and control experiment under an Ar atmosphere (PDF)

## References

[ref1] Kudo A. Springer Handbook of Inorganic Photochemistry “Heterogeneous Photocatalyst for CO_2_ Reduction”; Bahnemann D., Patrocinio A. O. T., Eds.; Springer International Publishing, 2022; pp 1369–1380.

[ref2] Hisatomi T.; Domen K. Introductory Lecture: Sunlight-Driven Water Splitting and Carbon Dioxide Reduction by Heterogeneous Semiconductor Systems as Key Processes in Artificial Photosynthesis. Faraday Discuss. 2017, 198, 11–35. 10.1039/c6fd00221h. 28272623

[ref3] Nishiyama H.; Yamada T.; Nakabayashi M.; Maehara Y.; Yamaguchi M.; Kuromiya Y.; Nagatsuma Y.; Tokudome H.; Akiyama S.; Watanabe T.; Narushima R.; Okunaka S.; Shibata N.; Takata T.; Hisatomi T.; Domen K. Photocatalytic Solar Hydrogen Production from Water on a 100-m^2^ Scale. Nature 2021, 598, 304–307. 10.1038/s41586-021-03907-3. 34433207

[ref4] Iizuka K.; Wato T.; Miseki Y.; Saito K.; Kudo A. Photocatalytic Reduction of Carbon Dioxide over Ag Cocatalyst-Loaded ALa_4_Ti_4_O_15_ (A = Ca, Sr, and Ba) Using Water as a Reducing Reagent. J. Am. Chem. Soc. 2011, 133, 20863–20868. 10.1021/ja207586e. 22087856

[ref5] Teramura K.; Wang Z.; Hosokawa S.; Sakata Y.; Tanaka T. A Doping Technique That Suppresses Undesirable H_2_ Evolution Derived from Overall Water Splitting in the Highly Selective Photocatalytic Conversion of CO_2_ in and by Water. Chem.—Eur. J. 2014, 20, 9906–9909. 10.1002/chem.201402242. 25044046

[ref6] Nakanishi H.; Iizuka K.; Takayama T.; Iwase A.; Kudo A. Highly Active NaTaO_3_-Based Photocatalysts for CO_2_ Reduction to Form CO Using Water as the Electron Donor. ChemSusChem 2017, 10, 112–118. 10.1002/cssc.201601360. 27874269

[ref7] Teramura K.; Tanaka T. Necessary and Sufficient Conditions for the Successful Three-Phase Photocatalytic Reduction of CO_2_ by H_2_O over Heterogeneous Photocatalysts. Phys. Chem. Chem. Phys. 2018, 20, 8423–8431. 10.1039/c7cp07783a. 29542742

[ref8] Yoshino S.; Takayama T.; Yamaguchi Y.; Iwase A.; Kudo A. CO_2_ Reduction Using Water as an Electron Donor over Heterogeneous Photocatalysts Aiming at Artificial Photosynthesis. Acc. Chem. Res. 2022, 55, 966–977. 10.1021/acs.accounts.1c00676. 35230087 PMC8988292

[ref9] Hori Y.; Wakebe H.; Tsukamoto T.; Koga O. Electrocatalytic Process of CO Selectivity in Electrochemical Reduction of CO_2_ at Metal Electrodes in Aqueous Media. Electrochim. Acta 1994, 39, 1833–1839. 10.1016/0013-4686(94)85172-7.

[ref10] Hori Y. Electrochemical CO_2_ Reduction on Metal Electrodes. In Modern Aspects of Electrochemistry; Vayenas C. G., White R. E., Gamboa-Aldeco M. E., Eds.; Springer: New York, 2008; pp 89–189.

[ref11] Kuhl K. P.; Hatsukade T.; Cave E. R.; Abram D. N.; Kibsgaard J.; Jaramillo T. F. Electrocatalytic Conversion of Carbon Dioxide to Methane and Methanol on Transition Metal Surfaces. J. Am. Chem. Soc. 2014, 136, 14107–14113. 10.1021/ja505791r. 25259478

[ref12] Nitopi S.; Bertheussen E.; Scott S. B.; Liu X.; Engstfeld A. K.; Horch S.; Seger B.; Stephens I. E. L.; Chan K.; Hahn C.; Nørskov J. K.; Jaramillo T. F.; Chorkendorff I. Progress and Perspectives of Electrochemical CO_2_ Reduction on Copper in Aqueous Electrolyte. Chem. Rev. 2019, 119, 7610–7672. 10.1021/acs.chemrev.8b00705. 31117420

[ref13] Nakagawa S.; Kudo A.; Azuma M.; Sakata T. Effect of Pressure on the Electrochemical Reduction of CO_2_ on Group VIII Metal Electrodes. J. Electroanal. Chem. 1991, 308, 339–343. 10.1016/0022-0728(91)85080-9.

[ref14] Kudo A.; Nakagawa S.; Tsuneto A.; Sakata T. Electrochemical Reduction of High Pressure CO_2_ on Ni Electrodes. J. Electrochem. Soc. 1993, 140, 1541–1545. 10.1149/1.2221599.

[ref15] Habisreutinger S. N.; Schmidt-Mende L.; Stolarczyk J. K. Photocatalytic Reduction of CO_2_ on TiO_2_ and Other Semiconductors. Angew. Chem., Int. Ed. 2013, 52, 7372–7408. 10.1002/anie.201207199. 23765842

[ref16] White J. L.; Baruch M. F.; Pander J. E.; Hu Y.; Fortmeyer I. C.; Park J. E.; Zhang T.; Liao K.; Gu J.; Yan Y.; Shaw T. W.; Abelev E.; Bocarsly A. B. Light-Driven Heterogeneous Reduction of Carbon Dioxide: Photocatalysts and Photoelectrodes. Chem. Rev. 2015, 115, 12888–12935. 10.1021/acs.chemrev.5b00370. 26444652

[ref17] Li X.; Yu J.; Jaroniec M.; Chen X. Cocatalysts for Selective Photoreduction of CO_2_ into Solar Fuels. Chem. Rev. 2019, 119, 3962–4179. 10.1021/acs.chemrev.8b00400. 30763077

[ref18] Ran J.; Jaroniec M.; Qiao S. Cocatalysts in Semiconductor-based Photocatalytic CO_2_ Reduction: Achievements, Challenges, and Opportunities. Adv. Mater. 2018, 30, 1704649 10.1002/adma.201704649. 29315885

[ref19] Li P.; Zhou Y.; Zhao Z.; Xu Q.; Wang X.; Xiao M.; Zou Z. Hexahedron Prism-Anchored Octahedronal CeO_2_ : Crystal Facet-Based Homojunction Promoting Efficient Solar Fuel Synthesis. J. Am. Chem. Soc. 2015, 137, 9547–9550. 10.1021/jacs.5b05926. 26194000

[ref20] Lu L.; Wang B.; Wang S.; Shi Z.; Yan S.; Zou Z. La_2_O_3_-Modified LaTiO_2_N Photocatalyst with Spatially Separated Active Sites Achieving Enhanced CO_2_ Reduction. Adv. Funct. Mater. 2017, 27, 1702447 10.1002/adfm.201702447.

[ref21] Wang Y.; Zhang Z.; Zhang L.; Luo Z.; Shen J.; Lin H.; Long J.; Wu J. C. S.; Fu X.; Wang X.; Li C. Visible-Light Driven Overall Conversion of CO_2_ and H_2_O to CH_4_ and O_2_ on 3D-SiC@2D-MoS_2_ Heterostructure. J. Am. Chem. Soc. 2018, 140, 14595–14598. 10.1021/jacs.8b09344. 30351926

[ref22] Iwase A.; Kato H.; Kudo A. The Effect of Alkaline Earth Metal Ion Dopants on Photocatalytic Water Splitting by NaTaO_3_ Powder. ChemSusChem 2009, 2, 873–877. 10.1002/cssc.200900160. 19731285

[ref23] Kato H.; Asakura K.; Kudo A. Highly Efficient Water Splitting into H_2_ and O_2_ over Lanthanum-Doped NaTaO_3_ Photocatalysts with High Crystallinity and Surface Nanostructure. J. Am. Chem. Soc. 2003, 125, 3082–3089. 10.1021/ja027751g. 12617675

