# Supplementary material for: CH4 Synthesis from CO2 and H2O of an Electron Source over Rh–Ru Cocatalysts Loaded on NaTaO3:Sr Photocatalysts
Source: J Am Chem Soc. 2023 Aug 21;145(37):20485–91. doi: 10.1021/jacs.3c06413 (PMC10515478; doi:10.1021/jacs.3c06413)
Supplement: Supplementary file 1 — ja3c06413_si_001.pdf [file ja3c06413_si_001.pdf]

Supporting Information

CH_4 Synthesis from CO_2 and H_2O of an electron source over Rh-Ru cocatalyst loaded on $NaTaO_3$:Sr photocatalyst

Wasusate Soontornchaiyakul,[a] Shunya Yoshino,[a] Tomoki Kanazawa,[b] Rie Haruki,[b] Dongxiao Fan,[b] Shunsuke Nozawa,[b] Yuichi Yamaguchi,[a,c] Akihiko Kudo[a,c]*

[a]Department of Applied Chemistry, Faculty of Science, Tokyo University of Science, 1-3 Kagurazaka, Shinjuku-ku, Tokyo 162-8601, Japan

[b]Institute of Materials Structure Science, High Energy Accelerator Research Organization, Tsukuba, Ibaraki 305-0801, Japan

[c]Carbon Value Research Center, Research Institute for Science & Technology, Tokyo University of Science, 2641 Yamazaki, Noda-shi, Chiba-ken, 278-8510, Japan

Email: a-kudo@rs.tus.ac.jp

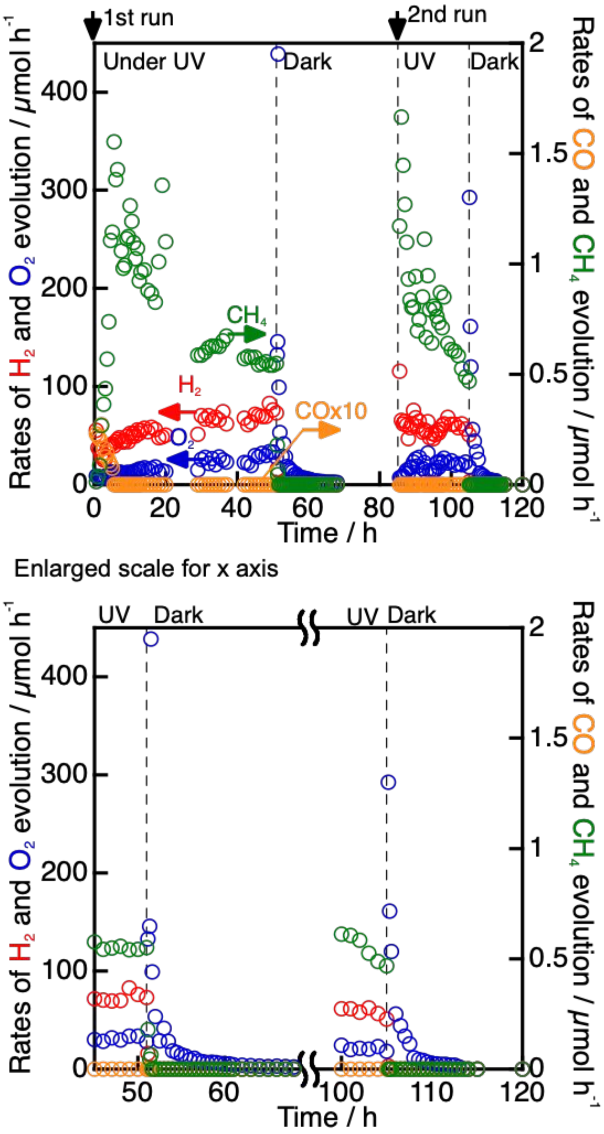

Figure S1. Production rate for photocatalytic CO_2 reduction using Rh(0.5 wt%)-Ru(0.375 wt%)/$NaTaO_3$:Sr(1%) photocatalyst under UV irradiation corresponding to Figure 1(d). Photocatalyst: 1.5 g, solution: water without any additives (350 mL, pH 4-5), flow gas: CO_2 (1 atm), light source: 400 W high pressure Hg lamp, and cell: inner-irradiation quartz cell.

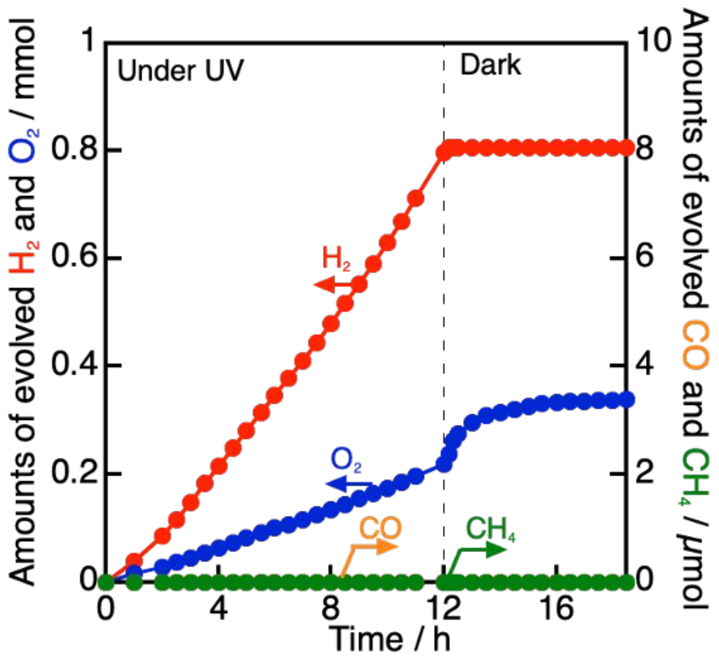

Figure S2. Photocatalytic water splitting using Rh(0.5 wt%)-Ru(0.375 wt%)/$NaTaO_3$:Sr(1%) photocatalyst under UV irradiation. Photocatalyst: 1.5 g, solution: water with H_2SO_4 (350 mL, pH 4), flow gas: Ar (1 atm), light source: 400 W high pressure Hg lamp, and cell: inner-irradiation quartz cell.
